# Sequential Treatment With Targeted and Immune Checkpoint Therapy in Patients With BRAF Positive Metastatic Melanoma: The Importance of Timing?

**DOI:** 10.3389/fmed.2019.00257

**Published:** 2019-12-17

**Authors:** Victoria Grätz, Detlef Zillikens, Hauke Busch, Ewan A. Langan, Patrick Terheyden

**Affiliations:** ^1^Department of Dermatology, University of Lübeck, Lübeck, Germany; ^2^Lübeck Institute of Experimental Dermatology, University of Lübeck, Lübeck, Germany; ^3^Institute of Cardiogenetics, University of Lübeck, Lübeck, Germany; ^4^Dermatological Science, University of Manchester, Manchester, United Kingdom

**Keywords:** melanoma, sequential treatment, targeted therapy, immunotherapy, BRAF mutation

## Abstract

**Background:** Immune checkpoint- and targeted therapy have dramatically improved the therapeutic landscape in the management of BRAF mutation positive metastatic melanoma. However, pending the results of clinical trials, not only is it currently unclear whether immune checkpoint- or targeted therapy should be commenced up front, but the optimal time for changing treatment, specifically to prevent resistance whilst maintaining disease control, is unknown.

**Methods:** We retrospectively identified eleven patients with BRAF V600 mutated metastatic melanoma who commenced targeted therapy between 11/2012 and 12/2017 in our center. In 5 cases the decision was made to “electively” switch to immune checkpoint therapy (elective group) following the development of a complete or partial response. In the remaining 6 cases the initial “reactive” switch was necessitated by disease progression or the development of intolerable side-effects (reactive group).

**Results:** Overall, the elective cohort had a more favorable course in terms of overall survival (1,003 vs. 827 days), and 80% of the patients remain alive, in contrast to 17 % of the patients in the reactive group. However, it should be borne in mind that multiple switches due to disease progression were undertaken and this undoubtedly also impacted upon overall survival.

**Conclusion:** Elective switching from targeted to immune checkpoint therapy was associated with a better outcome in terms of survival, at least in everyday clinical practice. It remains unclear whether the choice of initial therapy confers long–term survival and disease-control advantages and this should be addressed in prospective studies.

## Introduction

The therapeutic options for the management of metastatic melanoma in BRAF-mutated patients have improved dramatically with the development of targeted and immune checkpoint based therapies. The BRAF activating mutation is present in 40–50% of melanomas, providing an important therapeutic target that can be clinically exploited by inhibiting the MAPK/ERK signaling pathway (1). The combination of BRAF and MEK inhibition is associated with improved overall survival (OS) and may reduce the incidence of resistance (2, 3). Indeed, Schadendorf et al. reported that pooled analyses of clinical trials of dabrafenib and trametinib revealed a 3 year overall survival (OS) rate of 44% (4). Recently updated data revealed a 5 year OS of 34% (5). In patients with favorable prognostic factors, including a normal serum lactate dehydrogenase (LDH) level, the sum of lesion diameters <66 mm and metastases in <3 organs, the 3 year progression free survival (PFS) rate was 42% (4). Similarly, Hauschild et al. reported 3 year OS rates of 53.3 % in patients with favorable prognostic features treated with cobimetinib and vemurafenib in a retrospective analysis of data from 4 randomized clinical trials (6). In fact, targeted therapy leads to a favorable tumor microenvironment in melanoma, with increased CD8 positive T cell infiltration and PD-L1 expression, suggesting a potential synergistic effect with immunotherapy (7).

Pending the results of ongoing clinical trials, the optimal first-line treatment strategy remains unclear and is likely to remain patient- and tumor-specific. Given the rapid response to targeted therapy, accompanied by a dramatic decrease in overall tumor load, the decision to commence targeted therapy in patients with BRAF V600 mutations may be favored in the context of symptomatic disease and the presence of adverse prognostic markers, including raised serum LDH concentrations, ECOG performance status> 1, younger patients, and those with brain and/or metastases at multiple sites (8, 9). Typically, resistance to targeted therapy occurs after a median treatment time of 13 months. The use of immune checkpoint based therapies presents an important treatment option in the context of resistance to targeted therapies; the use of anti-programmed death protein (PD)-1 therapies is associated with an impressive overall response and level of disease control, albeit with a slower onset of action, but potentially a more durable effect (10, 11). Furthermore, the published data points toward a more favorable outcome in patients with brain metastases treated with combined immune checkpoint therapy with nivolumab and ipilimumab (12). At present, the decision as to whether to first employ targeted- or immune checkpoint therapy in patients with BRAF positive melanoma is reached after careful consideration of the overall disease burden, LDH levels, the presence of central nervous system metastases and clinician/individual patient preference. Several trials are currently examining the efficacy of sequenced targeted and immunotherapy (NCT02902029, NCT03235245) in order to establish whether sequential treatment represents a useful treatment strategy to deliver and sustain disease control.

Pending the results from these trials, we describe our clinical experience in 11 patients with melanoma stage IV, who were initially treated with targeted therapy and switched to immune checkpoint therapy (i) electively (based on partial or complete response and to prevent resistance) or (ii) in response to disease progression, in a sequential order.

## Patients and Methods

**Figure 1:**
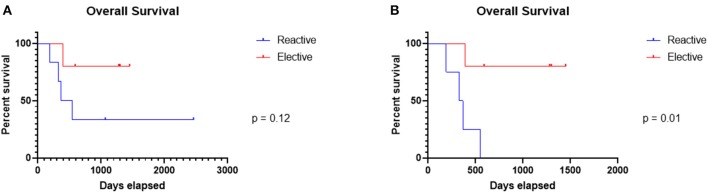
Overall survival in the elective and reactive cohorts. Whilst there was no significant difference in overall survival between the cohorts **(A)**, exclusion of the outlier patient, the only patient to have received chemotherapy prior to the switch from targeted to immunotherapy, and the patient who committed suicide, revealed a significant (*p* = 0.01) difference in overall survival **(B)**.

We retrospectively analyzed the clinical course of 11 patients with BRAF V600 mutation positive malignant melanoma who commenced targeted therapy between 11/2012 and 12/2017 in our center (Figure 1). A partial or complete radiological response was required in order to justify electively switching treatment from targeted to immune therapy to prevent the development of resistance. When staging examinations (CT/MRI) revealed disease progression the treatment was switched from targeted to immune therapy in a reactive manner due to an inadequate treatment response. The retrospective analysis was approved by the University of Luebeck's ethics committee (19-117A). Graph Pad Prism (Version 8.0.2) was used for the survival analyses and survival curves were compared with log rank (Mantel-Cox) tests. A *p* < 0.05 was considered significant.

## Results

### Elective Switching From Targeted to Immunotherapy Is Associated With Improved Overall Survival

In order to ascertain the clinical course and calculate the overall survival of patients who were treated with sequential targeted therapy followed by immunotherapy a retrospective analysis of the electronic case notes was performed. Patients were retrospectively assigned to an “elective” or “reactive” cohort depending on whether therapy was switched electively on the basis of a radiological partial or complete response or reactively due to disease radiographic disease progression and/or intolerable side-effects (see Tables 1–3). There were no significant differences between the baseline characteristics of the groups in terms of age, baseline lactate dehydrogenase, and serum S100 concentrations.

**Table 1:** Patient characteristics in both the elective and reactive cohorts.

**Patient** ** characteristics**	**Elective** ** group**	**Reactive** ** group**	***p* value**
**Sex**			
Male	4	5	
Female	1	1	
**Age**			
Range (years)	39–67	24–75	
Mean	51.4	54.7	0.75
**Overall survival**			
Range (days)	393–1451	192–2462	
Mean	1002.8	827.2	0.69
**Primary tumor site**			
Unknown primary	2	0	
Back	1	1	
Leg	2	3	
Head/Neck	0	2	
Mean S100 prior to targeted therapy μg/l	0.41	1.06	0.30
Mean LDH prior to targeted therapy U/l	244.8	328.5	0.33

**Table 2:** Switching from targeted to immunotherapy when a partial or complete response is achieved.

**Sex**	**Age**	**Primary tumor**	**Rx 1**	**S100 Normal value <0.11 μg/l**	**LDH Normal value <250 U/l**	**Pre-Switch response**	**Duration (days)**	**Rx 2**	**S100 2**	**LDH 2**	**Duration 2**	**Rx 3**	**S100 3**	**LDH 3**	**Duration 3**	**Rx 4**	**S100 4**	**LDH 4**	**Duration 4**	**Rx 5**	**S100 5**	**LDH 5**	**Duration 5**	**Rx 6**	**S100 6**	**LDH 6**	**Duration 6**	**Rx 7**	**S100 7**	**LDH 7**	**Duration 7**	**OS (days)/Status**
M	50	MUP	Vem/Cobi	0.09	207	PR	152	Nivolumab/Ipilimumab	0.08	302	433	Vem/Cobi	0.99	273	356	Nivolumab 3 mg/kg x 1	0.11	310	215	Enco/Bini	0.30	233	271									1299 - Alive
								4 Cycles				Thrombocytopenia				Nivolumab 480 mg																
								Autoimmune hepatits grade 3				Prednisolone 1 mg/kg				7 Cycles																
								Prednisolone 2 mg/kg																								
								Cellcept 1 g b.d.																								
								Nivolumab 3 mg/kg monotherapy																								
								3 Cycles																								
F	43	Nodular Melanoma	Dab/Tram	0.18	179	CR	210	Nivolumab/Ipilimumab	0.05	215	43	Dab/Tram	0.07	189	80	Nivolumab 3 mg/kg	0.05	183	107	Vem/Cobi	0.08	178	10	Ipilimumab 3 mg/kg	0.08	172	54	Dab/Tram	0.44	174	655	1280 - Alive
		Left thigh						1 Cycle								8 Cycles				Sepsis				Nivolumab 1 mg/kg				Sarcoidosis				
								Autoimmune thyroiditis grade 3								Radiotherapy				Drug-induced exanthem				4 Cycles				Cellultis				
								Mumps infection																				Exzision of a subcutaneous metastasis				
								Exzision of a subcutaneous																								
								metastasis																								
M	67	Melanoma	Vem (2 × 960 mg)	1.43	407	PR	54	Ipilimumab 3 mg/kg	0.05	181	78	Dab/Tram	1.66	343	196	Nivolumab 3 mg/kg	0.41	308	30													393 - Dead
		Left thigh						4 Cycles								2 Cycles																
M	39	SSM	Vem/Cobi	0.29	262	PR	303	Nivolumab/Ipilimumab	0.05	327	54	Vem/Cobi	0.04	204	161	Pembrolizumab 2 mg/kg	0.05	312	168	Ipilimumab/Nivolumab	0.04	191	544	Enco/Bini	0.05	218	13					1451 - Alive
		Back						4 Cycles								8 Cycles				2 Cycles Pembrolizumab												
								Autoimmune thyroiditis grade 3								Radiotherapy				8 Cycles 2 mg/kg												
								Autoimmune hepatitis grade 2												Pembrolizumab 200 mg												
								Prednisolone 1 mg/kg												11 Cycles fortnightly												
								Neutropenia												Pembrolizumab 400 mg												
								Nivolumab 3 mg/kg monotherapy												2 Cycles in 3 week intervals												
								6 Cycles												Radiotherapy												
M	58	MUP	Vem/Cobi	0.06	169	CR	188	Nivolumab/Ipilimumab	0.09	207	96	Enco/Bini	0.07	193	293																	591 - Alive
								4 Cycles				Radiotherapy																				
								Colitis grade 3																								
								Prednisolone 1 mg/kg																								

*The clinical course in patients electively switched from targeted to immune therapy based on the clinical response*.

*Dabra/Tram, Dabrafenib/Trametinib; Enco/Bini, Encorafenib; Binimetinib; LDH, lactate dehydrogenase; MUP, melanoma of unknown primary; SSM, superficial spreading melanoma; CR, complete response; PD, progressive disease; PR, partial response; Vem/Cobi; Vemurafenib/Cobimetinib*.

**Table 3:** Switching from targeted to immunotherapy due to disease progression or intolerable side-effects.

**Sex**	**Age**	**Primary tumor**	**Rx 1**	**S100 1**	**LDH 1**	**Pre-Switch response**	**Duration**	**Rx 2**	**S100 2**	**LDH 2**	**Duration 2**	**Rx 3**	**S100 3**	**LDH 3**	**Duration** ** 3**	**Rx 4**	**S100 4**	**LDH 4**	**Duration 4**	**Rx 5**	**S100 5**	**LDH 5**	**Duration 5**	**Rx 6**	**S100 6**	**LDH 6**	**Duration 6**	**Rx 7**	**Rx 8**	**Rx 9**	**Rx 10**	**Rx 11**	**OS (days)/Status**
M	75	SSM	Dab/Tram	0.37	267	Therapy changed due to side effects:	85	Vem/Cobi	0.17	371	219	Nivolumab	0.06	203	91	Nivolumab/ Ipilimumab	0.11	223	115														547 - Dead
		Right Thigh										3 mg/kg 7 Cycles				2 Cycles																	
						Recurrent Pyrexia										Radiotherapy																	
																Colitis Grade 3																	
																Prednisolone 1 mg/kg																	
F	45	Nodular Melanoma	Dab then Vem	1.17	258	Therapy changed due to side effects:	101	Dacarbazine	0.156	187	71	Ipilimumab	3.03	383	79	Nivolumab 3 mg/kg	4.11	366	196	Vem	8.49	634	707	Ipilimumab 3 mg/kg	197	0.06	92	Nivolumab	Vem/ Cobi	Nivolumab	Vem/ Cobi	Ipilimumab/Nivolumab	2462 - Alive
		Back						250 mg/m^2^ over 5 days				3 mg / kg				14 Cycles				Vem/Cobi				2 Cycles						480 mg		3 mg/kg and 1 mg/kg	
						Retinitis Serosa		3 Cycles				4 Cycles				Radiotherapy								Radiotherapy						2 Cycles		4 Cycles	
M	24	SSM	Enco/Bini	3.13	575	PD	224	Nivolumab/ Ipilimumab	0.64	420	23	Dab/Tram	2.51	755	32	Dacarbazine 1000 mg/m^2^	3.98	620															368 - Dead
		Back						2 Cycles				Radiotherapy				2 Cycles																	
								Radiotherapy																									
M	54	Nodular Melanoma	Dab/Tram	0.12	214	PD	62	Nivolumab 3 mg/kg	0.16	308																							192 - Dead
		Back						5 Cycles																									
								Radiotherapy																									
M	56	Nodular Melanoma	Dab/Tram	N/A	467	PD	318	Nivolumab/ Ipilimumab	0.03	195																							329 - Dead
		Neck																															
M^*^	74	Nodular Melanoma	Enco/Bini	0.53	190	PD	284	Pembrolizumab	0.03	179	203	Cobi/Vem	173	0.04	69	Pembrolizumab	0.04	168	456														1065 - Dead
		Right ear						10 Cycles				Surgery				20 Cycles																	
												Radiotherapy																					

*The clinical course is detailed in patients switched from targeted to immune therapy due to disease progression or intolerable treatment related side-effects*.

* *The patient committed suicide during the study period*.

*Dabra/Tram, Dabrafenib/Trametinib; Enco/Bini, Encorafenib; Binimetinib; LDH, lactate dehydrogenase; MUP, melanoma of unknown primary; SSM, superficial spreading melanoma; CR, complete response; PD, progressive disease; PR, partial response; Vem/Cobi; Vemurafenib/Cobimetinib*.

As expected with two small cohorts, there was no significant difference in terms of the length of overall survival between the groups. However, it is worth noting that the clinical course in Patient 2 (reactive group) differed markedly from that of the other patients in the cohort. Not only did the patient initially receive monotherapy with BRAF inhibition, but the patient also received chemotherapy with dacarbazine prior to being switched to ipilimumab. The patient was only then switch to combined targeted therapy due to disease progression.

Interestingly, exclusion of the outlier patient and the patient who committed suicide in the reactive group revealed a significant (*p* = 0.01) difference in overall survival.

Moreover, the average length of overall survival in the elective group was 1,003 days compared to 827 days in the reactive group. At last follow up, 83% of the patients in the reactive cohort had died, including one patient who committed suicide whilst only one patient (20%) in the elective group had died.

## Discussion

An overall survival benefit of elective switching from targeted to immunotherapy could be demonstrated in 4 out of 5 cases, with a maximal OS of up to almost 4 years. In each of these patients the decision to switch from targeted therapy to immunotherapy was made electively at the time of complete or partial response, given the overall clinical response and to prevent the development of treatment resistance.

Initial treatment with targeted therapy may be favored in patients with a BRAF V600 mutation in the context of a large tumor burden and adverse prognostic factors (including increased LDH) in whom rapid disease control is of paramount importance. Whether initial treatment with checkpoint immunotherapy in patients with BRAF mutations, provides any long-term and durable therapeutic advantages over targeted therapy remains the subject of intense investigation. As outlined by Luke et al. the decision to initiate targeted therapy may be favored when rapid disease control and/or immune-priming effects are required, whereas increased LDH and avoidance of resistance may favor initial checkpoint therapy (13).

Ackerman et al. analyzed the outcome of 274 patients treated with immunotherapy prior to (*n* = 32) or after (*n* = 242) BRAF inhibition. This retrospective study reported that prior treatment with targeted therapy did not negatively influence the response to subsequent immunotherapy with ipilimumab. However, outcomes for patients treated with ipilimumab following BRAF inhibition were poor (14). It should be noted that this was retrospective study and at the time the only licensed immunotherapy was monotherapy with ipilimumab. Aya et al. (8) failed to uncover any differences in overall survival in a small retrospective cohort study specifically comparing targeted then immune therapy and vice versa. However, results from prospective trials examining sequential therapy, e.g., NCT02224781 or SECOMBIT (NCT02631447) with additional checkpoint inhibitors, are eagerly anticipated.

The combination of targeted therapy and immunotherapy, either in a parallel or sequential manner, could theoretically lead to enhanced anti-tumor responses, reflected in durable responses and prolonged survival. In fact, the length of response to BRAF/MEK inhibition may be key. For example, Ascierto et al. reported that in patients who responded to BRAF/MEK inhibition for over 6 months, the overall response rate to subsequent anti-PD1 therapy was 34% (15). In contrast, when patients benefits for <6 months, the overall response rate to subsequent anti-PD1 therapy was only 15%. This sits well with the evidence that BRAF inhibition can improve the efficacy of PD-1 blockade via changes in the tumor microenvironment (10, 15, 16). In fact, BRAF/MAPK targeted therapy can alter the immune environment within 2 weeks (16), resulting in elevated PD-L1 expression for up to 3 months (17). It is however important to bear in mind that these changes may be temporary, perhaps opening up a therapeutic window in which the potential benefits from switching to immune therapy can be harnessed. Other authors have reported complete and durable remission using finite courses of BRAF inhibition following failure to respond to immunotherapy (18). However, these observations are based on small case series. Most recently concern has been raised about the tolerability and side effect profile of BRAF/MEK inhibition after anti-PD-1 therapy (19). Indeed, the authors speculated that the increased incidence of treatment interruptions may impact upon the rates of OS.

In contrast to this observation (19), it has been reported that initial anti-PD-1 therapy in patients with BRAF mutations may be associated with improved overall survival when compared to patients initially treated with targeted therapy (20). Despite being a multi-centric analysis, these data were again retrospective.

Overall, we witnessed fewer treatment related toxicities in the reactive switch group. Given that the development of toxicities is associated with an improved response to immunotherapy (21), the lack of side-effects correlated well with the lack of disease control and overall poorer prognosis.

A major limitation of our case series is its retrospective nature. Decisions on which targeted therapies and which immune checkpoint therapies were administered (anti-PD-1 monotherapy vs. combined anti-PD-1 and anti-CTLA-4 therapy) were taken by the multi-disciplinary tumor board. These decisions were based on tumor factors (tumor activity and overall disease burden) and patient factors (including relevant co-morbidities). Whilst the various treatment combinations and retrospective nature of the analysis could be interpreted as weaknesses, it should nevertheless be borne in mind that the data reflect the “real-life” clinical management of metastatic melanoma.

Summarizing the results of sequential therapy in our cohort, it is reasonable to conclude that switching between targeted and immune checkpoint therapy, and vice versa, which can be complemented by radiotherapy of brain metastases, may be associated with improved long-term survival, even in patients with an extensive disease burden. This treatment strategy may be useful strategy to prevent the development of resistance to MEK/BRAF inhibition. However, given the historical context of our retrospective analysis which meant that all patients were initially treated with targeted therapy, no conclusions can be drawn on which upfront treatment strategy is best.

In the absence of evidence-based clinical data, the decision to switch between targeted and immune checkpoint based therapy and the importance of timing in terms of switching treatment modality remains a clinical conundrum (22). Indeed, with increasing rates and duration of overall survival, the specific contribution of the upfront therapy and therapy switches to any survival benefit is difficult to ascertain. Moreover, progression-free survival is also of limited benefit to measure treatment efficacy, given that targeted therapy treatment may be switched to immune therapy “electively” before tumor progression to prevent the development of resistance. Identifying the optimal time to switch therapy, depending on the duration and extent of the response to treatment, remains to be determined definitively. However, the publication of non-selected real-life clinical data reporting the long-term efficacy and tolerability of sequential therapy may help inform clinical practice until the definitive results from the on-going, prospective, and multi-center clinical trials are available.

## Data Availability Statement

The datasets generated for this study are available on request to the corresponding author.

## Ethics Statement

The study was reviewed and approved by University of Lübeck Ethics Committee (19-117A).

## Author Contributions

VG provided the patient details. VG, EL, and PT wrote the manuscript. HB provided biostatisical advice. All co-authors critically reviewed and edited the manuscript.

### Conflict of Interest

EL has received travel support (Curevec, Novartis) and speaker's honoraria (Novartis) and also participated in advisory boards from Novartis. PT has received speaker's honoraria from BMS, Novartis, and Roche, consultant's honoraria from BMS, Merck, Novartis, Sanofi, and Roche, and travel support from BMS, Pierre-Fabre and Roche. The remaining authors declare that the research was conducted in the absence of any commercial or financial relationships that could be construed as a potential conflict of interest.
